# Dementia Care: Addressing Pain and Maximizing Comfort

**DOI:** 10.1093/geroni/igab046.1120

**Published:** 2021-12-17

**Authors:** Deepa Vinoo

**Affiliations:** NYC Health Hospitals/Coler, Roosevelt Island, New York, United States

## Abstract

Pain is common in older people who have Dementia, and is associated with a number of chronic and acute conditions. There is evidence that as many as 83% of nursing home residents experience pain that often goes unrecognized or inappropriately treated. Pain has a powerful effect on mood, sleep quality, functional ability, and overall quality of life. Rejecting care due to pain is very common among patients with Dementia. An association between pain and increased agitation has been noted, Significant reduction of agitation and psychotropic usage have been demonstrated by pain treatment in patients with moderate to severe dementia. This project was conducted in six memory care units with 150 residents at 815 bedded long-term geriatric care facility. All residents in memory care units from May 2018 to December 2019 were individually assessed for pain management, rejection of care, usage of psychotropics, falls and physical altercations. Trained interdisciplinary staff to evaluate pain by using PAIN AD. Educated interdisciplinary team on pharmacological and non-pharmacological pain management, and Pain management has improved from 40% to 90%, Rejection of care reduced from 80% to 30%. Usage of antipsychotics reduced by 12%. Falls reduced from 12% to 2%. Physical altercations reduced to zero. Staff call out due to work related injury significantly reduced. Staff verbalized improved job satisfaction and increased morale.

